# A Nationwide, Population-based Cohort Study on Potential Autoimmune Association of *Ménière* Disease to Atopy and Vitiligo

**DOI:** 10.1038/s41598-019-40658-8

**Published:** 2019-03-13

**Authors:** Hyung Jin Hahn, Sang Gyu Kwak, Dong-Kyu Kim, Jong-Yeup Kim

**Affiliations:** 1 Department of Dermatology, College of Medicine, Konyang University, Daejeon, Republic of Korea; 2 School of Medicine, Department of Medical Statistics, Catholic University of Daegu, Gyeongsan, South Korea; 3 Department of Otorhinolaryngology-Head and Neck Surgery, Chuncheon Sacred Heart Hospital, Hallym University College of Medicine, Chuncheon, Republic of Korea; 4 Institute of New Frontier Research, Hallym University College of Medicine, Chuncheon, Republic of Korea; 5 Department of Otorhinolaryngology–Head and Neck Surgery, College of Medicine, Konyang University, Daejeon, Republic of Korea; 6 Department of Biomedical Informatics, College of Medicine, Konyang University, Daejeon, Republic of Korea; 7 Myunggok Medical Research Institute, College of Medicine, Konyang University, Daejeon, Republic of Korea

## Abstract

*Ménière* disease (MD), an idiopathic disorder of sensorineural hearing loss and vertigo, shares many similarities with two common skin conditions, atopic dermatitis (AD) and vitiligo. Recent studies have suggested that MD may be related to or triggered by autoimmune conditions, notably Hashimoto thyroiditis and alopecia areata. These evidences led to the authors contemplating the possibility of immunological bridge between MD and the two skin conditions. The authors have tested this hypothesis with population-based cohort from the National Health Insurance Service Database of Korea. A cohort of 1.1 million patients was extracted from the database. Using *χ*^2^ tests, prevalence of the two skin disorders in relation to MD status was analysed. In MD patients, the odds ratios for having concurrent AD and vitiligo were 0.717 (95% CI, 0.535–0.962, *p* = 0.026) and 2.149 (95% CI, 1.396–3.308, *p* = 0.001), respectively. Females and older patients were more than twice likely to be affected by the two skin conditions. The relationship between vitiligo and MD was significant in younger subgroup only. Socio-economic subgroup analysis revealed the observed patterns are primarily a middle-upper class phenomenon. Uncertainty regarding temporal sequence of onset, and lack of detail on disease severity and subtype kept the study from more refined conclusion. In concluding, AD and vitiligo might be linked to MD through T_*reg*_-driven action of cellular immunity, but further big data-based investigations must follow.

## Introduction

After one-and-a-half centuries since Prosper Ménière first described the mysterious, paroxysmal attacks of dizziness he and his associates came to dub *glaucome de l’oreille interne* (*Fr*., “glaucoma of the inner ear”), Ménière disease still by and large remains in the dark^1^. Today, Ménière disease (MD) sits atop the list of a peculiar group of sensorineural hearing loss, sometimes labelled *immune-mediated inner ear diseases*^2^ (IMIED). Vertigo, which is always accompanied by twitching of the eye (*nystagmus*), ear ringing (*tinnitus*) and fullness of the ear (aural impaction) are often touted as the classic *triad* of symptoms, but the presentation can be diverse, and some believe MD lies on a continuum between certain, polar extremes of sensorineural hearing loss^3^. It has been suspected for some time that the crux of the MD aetiology is autoimmunity, while viral infection and allergic sensitisation are implicated as other likely perpetrators^4,5^. Regardless of how it is instigated, the prolonged and repetitive assault that follows precipitates in vestibular fibrosis, and this in turn causes endolymphatic fluid to build up within the endolymphatic sac (ELS). Although this *endolymphatic hydrops* (ELH) is undoubtedly the centrepiece of the MD pathology, whether impaired endolymphatic flow is directly responsible for the clinical symptoms, or is merely an epiphenomenon, is not likely to be settled in a near future^6^.

Intriguingly, the vertiginous disorder features several unmistakable parallels to atopic dermatitis (AD) and vitiligo-two of the most common skin diseases of the contemporary times; as with MD, there is apparently a strong overtone of autoimmune diathesis in the pathophysiology. Like MD, AD and vitiligo are idiopathic entities, in which a complex array of interplays between genetic predisposition and environmental factors fuel the continuing process of skin inflammation and depigmentation^7,8^. They often take on a protracted, debilitating course, interposed by bouts of remissions and exacerbations^9^. Also, sensorineural system is implicated as one of contributing factors in all three conditions^10–12^, albeit to differing extent and by diverse mechanisms. Finally, through varying modes of delivery, corticosteroid forms an important pillar of management strategy.

In recent times, there has been ongoing attempts to establish potential association between the chronic inner ear condition and some of the more common autoimmune diseases. These studies have shown that MD may be related to or triggered by autoimmune conditions, notably Hashimoto thyroiditis^13^ and alopecia areata^14^. Extrapolating from these findings, the authors hypothesized that there must exist an immunological nexus linking MD and the two immune-mediated skin conditions. The authors have approached the conjecture using a population-based cohort from the National Health Insurance Service of Korea database.

## Results

### Baseline characteristics

Baseline demographic information is summarily given in Table 1. The whole cohort consisted of 1,113,656 individuals, with nearly equal sex distribution (M:F = 50.1:49.9). Geographically, the highest proportion of the cohort was drawn from Seoul and *Gyeonggi* Province, a metropolitan area surrounding the capital city (at around 21% apiece). Other major cities and their metropolitan provinces of the country were also evenly represented. In the Republic of Korea, each eligible citizen is covered through either one of two national health insurance plans, *i.e*., employee-insured or self employee-insured. Otherwise, one may be eligible for the Korean MedicAid program. For the purpose of subgroup analysis, the cohort was regrouped into younger and senile sides. The cut-off used was 65 years, reflecting the peak age of onset for MD^15^. The cohort was divided into ten income brackets (deciles), and then regrouped as *lower* (brackets 1 through 4), *middle* (brackets 5 through 7), or *upper* (brackets 8 through 10) income tiers. The study cohort was also divided from grade of 0 to 6 according to the extent of their disability, if present. For the entire cohort, “baseline” prevalence of MD, AD, and vitiligo was computed at 0.78%, 0.72% and 0.11%, respectively.

**Table 1:** Baseline characteristics. *Abbreviations*-*MD*, Ménière disease. *AD*, atopic dermatitis.

Variable		*n*	%
Total		1,113,656	100.0
Sex	Male	558,186	50.12
	Female	555,470	49.88
Age groups	≤65 years	1,037,231	93.14
	>65 years	76,425	6.86
Income Tiers	Lower (0~4)	326,641	29.33
	Middle (5~7)	356,257	31.99
	Upper (8~10)	430,758	38.68
Disability Grade	Normal (Grade 0)	1,087,242	97.63
	Moderate (Grade 1 & 2)	8,943	0.80
	Severe (Grade 3 to 6))	17,471	1.57
MD	No	1,104,991	99.22
	Yes	8,665	0.78
AD	No	1,105,616	99.28
	Yes	8,040	0.72
Vitiligo	No	1,112,385	99.89
	Yes	1,271	0.11

### Prevalence of AD in relation to MD status

Of the 8,620 MD individuals, 45 had concurrently been diagnosed with AD (0.52%). In contrast, the prevalence of AD in non-MD individuals (1,096,996 in total) was 0.72% (7,995 persons). The odds ratio (OR) was 0.717 (95% CI, 0.535–0.962, *p* = 0.026). MD females were more than twice likely to be affected by AD than the male cohort (Adjusted OR = 2.243, 95% CI, 2.142–2.349, *p* < 0.001). In addition, MD individuals over 65 years of age were 2.5 times more likely to be affected by AD compared to their younger counterparts (Adjusted OR = 2.486, 95% CI, 2.347–2.633, *p* < 0.001, Table 2).

**Table 2:** Prevalence of atopic dermatitis and vitiligo by *Ménière* disease status.

Variable		Crude			Adjusted^†^		
		OR	95% CI	*p*-value	OR	95% CI	*p*-value
AD	No	1	—	—	1	—	—
	Yes	0.717	0.535, 0.962	0.026*	0.751	0.560, 1.008	0.056
sex	Male				1	—	—
	Female				2.243	2.142, 2.349	<0.001*
age	≤65				1	—	—
	>65				2.486	2.347, 2.633	<0.001*

*Abbreviations*-*AD*, atopic dermatitis. *OR*, odds ratio. *CI*, confidence interval. *p < 0.05.

### Prevalence of vitiligo in relation to MD status

For vitiligo, 21 of the 8,644 MD individuals were also affected with the condition (0.24%). The prevalence of vitiligo in non-MD individuals was 0.11% (1,250 of 1,103,741). Both findings were statistically significant (*p*-values of 0.025 and 0.000, respectively). The odds ratio was 2.149 (95% CI, 1.396–3.308, *p* = 0.001). As in the case with AD, MD females were twice more likely to be affected by vitiligo (Adjusted OR = 2.242, 95% CI, 2.140–2.347, *p* < 0.001), and older individuals with MD were roughly 2.5 times more likely to be affected by vitiligo (Adjusted OR = 2.491, 95% CI, 2.352–2.639, *p* < 0.001, Table 3).

**Table 3:** Relationship between *Ménière* disease and vitiligo. *Abbreviation*-*OR*, odds ratio. *CI*, confidence interval.

Variable		Crude			Adjusted^†^		
		OR	95% CI	*p*-value	OR	95% CI	*p*-value
Vitiligo	No	1	—	—	1	—	—
	Yes	2.149	1.396, 3.308	0.001*	2.053	1.332, 3.165	0.001*
sex	Male				1	—	—
	Female				2.242	2.140, 2.347	<0.001*
age	≤65				1	—	—
	>65				2.491	2.352, 2.639	<0.001*

^†^Adjusted by sex (Male & Female) and age (≤65 & >65).

*Statistically significant for *p* < 0.05.

### Socioeconomic subgroups

*χ*^2^ test revealed that the pattern of MD-vitiligo relationship was valid only in the “middle” income tier (brackets 5 through 7, *p* = 0.000). On the other hand, the lower MD prevalence in AD patients was seen only in the “upper” tier (brackets 8 through 10, *p* = 0.037). Other relationships were not statistically significant (Table 4).

**Table 4:** Subgroups analysis-socioeconomic status.

Brackets	Skin disease		MD, *n* (%)		*p*-value
			No	Yes	
0~4	AD	No	322,420 (99.47)	2,505 (99.48)	0.949
		Yes	1,703 (0.53)	13 (0.52)	
	Vitiligo	No	323,832 (99.91)	2,514 (99.84)	0.250
		Yes	291 (0.09)	4 (0.16)	
5~7	AD	No	351,178 (99.26)	2,465 (99.52)	0.144
		Yes	2,602 (0.74)	12 (0.48)	
	Vitiligo	No	353,424 (99.90)	2,468 (99.64)	0.000*
		Yes	356 (0.10)	9 (0.36)	
8~10	AD	No	423,398 (99.14)	3,650 (99.46)	0.037*
		Yes	3,690 (0.86)	20 (0.54)	
	Vitiligo	No	426,485 (99.86)	3,662 (99.78)	0.218
		Yes	603 (0.14)	8 (0.22)	

*Abbreviations*-*AD*, atopic dermatitis. *MD*, Ménière disease. *p < 0.05.

### Disability

MD-AD/vitiligo relationship was also analysed by disability status. *χ*^2^ analysis yielded virtually the same results for individuals without disability as the whole cohort (*p*-values of 0.026 and 0.000 for AD and vitiligo, respectively). Interestingly, there was a reversal of pattern in the subgroup with moderate (grade 1 & 2) disability, with a five-fold increase in AD prevalence in MD patients (1 of 56 *versus* 22 of 8,864 in non-MD patients; *p* = 0.025).

## Discussion

Although the notion of immune-mediated inflammation in idiopathic, sensorineural hearing loss has persisted for over six decades^16^, little progress has since been made on the nature of the participant cellular components and the mechanism through which they interact to bring about inflammatory reactions. That said, available evidences have pointed to ELS as a key immunologic interface^17,18^. The labyrinthine sac is believed to harbour inner ear antigens of various molecular weights^19^, which can invoke autoimmune response (type II hypersensitivity) from the host defense^20^. Also, it has been suggested that the structure exhibits high affinity for circulating immune complex^21^, and the resulting immunologic reaction (type III hypersensitivity) breaches the permeability barrier, thus leading to ELH. In the light of all this, the most likely common denominators between MD and AD look to be cellular immunity, and particularly, the regulatory arm of T helper cells (T_*reg*_), along with allergic sensitisation. Given that the onset of AD generally precedes MD over the course of a lifespan (AD peaks in the second or third decade of life; MD in the seventh^22,23^), it appears that personal history of the former (or AD diathesis) lowers the risk of the latter. This apparent, “protective” effect of AD may be in explained in terms of changing T_*reg*_ *milieu*; while the vitality of T_*reg*_ in AD pathogenesis is hardly challenged, how the actual cell content or count changes is a subject of ongoing contention^24^. This is because T_*reg*_ are a remarkably heterogenous group of lymphocytes with widely variable functional capacities^25^. It appears plausible that T_*reg*_ somehow acquire a lasting, suppressive capabilities over the duration of AD, reconfiguring the T-cell microenvironment in such way that it deters emergence of autoimmune diseases in later life. Alternatively, this observation may be elaborated with tenets of the “hygiene” hypothesis^26^; the chronic inflammatory process of AD allows the lymphocytes to saturate autoantigens, hence reducing the probability of potential future autoimmune reactions. By the same token, the role of allergy in AD (especially extrinsic type) appears to come into action when antigen-specific lymphocytes deplete available allergens, lowering the chance of allergen-T helper cell contact at later period (*i.e*., antigenic “competition”^27^). The diametrical influences of MD on the prevalence of AD and vitiligo were found to be a middle-upper class phenomenon, and this may be another indication that the interrelationship obeys the hygiene theory. Meanwhile, the twofold increase in MD prevalence in vitiligo individuals may also be narrated from qualitative and quantitative changes in T_*reg*_ population; loss of melanocytes seen in vitiligo is mainly due to the action of CD8^+^ cytotoxic T cells (T_c_) which are in turn kept at bay by T_*reg*_^28^. Destruction of the T_*reg*_ by autoimmune process leads to widespread activation of the effector T_c_ without any backpedalling mechanism, facilitating the depigmentation process. The compromise in T_*reg*_ number and function is likely to set up a “breeding ground” for development of secondary autoimmune conditions, such as MD, at later stage of life. The purported importance of T_*reg*_ in MD pathogenesis is circumlocutorily supported by (1) increased natural killer (NK) cell activity in MD patients^29^ (NK cells are under suppressive regulation of T_*reg*_, particularly in the setting of autoimmunity^30^), and (2) the fact that T_*reg*_ are known to increase over the course of ageing process^31^, which might in turn be supported by the lack of association between vitiligo and MD in the senile group.

The present investigation is not without its limitations. First, the cross-sectional nature of the study meant that these new findings were built on the premise of the skin conditions preceding MD in onset. The validity of this assumption is difficult to ascertain since the exact prevalence and onset of MD tend to fluctuate from one report to another. Second, lack of information regarding disease severity and subtype had impeded more detailed analysis, which would have allowed the authors to propose more elaborate disease mechanism. Third, it was revealed by subgroup analysis that the relationship between the skin conditions and MD was statistically significant only for the middle-upper economic class cohort and the individuals *without* disability. In fact, prevalence of the three diseases was inversely related to both income level and the extent of disability (not shown in the figures). Although this study was based on a one-million strong, population-based cohort in which statistical power is hardly an issue and selection bias less of a concern (that is, in comparison to hospital records), it might not have been completely free from the clutches of “accessibility” bias (only patients with adequate income and leisure afford visit to dermatologists or otorhinolaryngologists).

## Conclusions

The present study has attempted to come up with a potential common thread between MD and two highly prevalent cutaneous conditions-atopic dermatitis and vitiligo from an autoimmune perspective. Notwithstanding its shortcomings, it allowed the authors to glimpse through the underlying patho-mechanism of three well known immune-mediated conditions, with some unique insights and perspectives. While all three diseases are still very much eluding investigators on every corner of the globe, further studies, using more sophisticated databases, would enable us to build upon this ground and yield more refined conclusions, including therapeutic implications.

## Methods

### Database (DB)

All study conduct adhered to the tenets of the Declaration of Helsinki. This study used KNHIS-NSC data (NHIS-2018-2-142), made by National Health Insurance Service (NHIS) and was approved by the Institutional Review Board of *Hallym* Medical University *Chuncheon* Sacred Hospital (IRB No. 2016-52). The need for written informed consent was waived because the KNHIS-NSC data set consisted of deidentified secondary data for research purposes.

The NHIS is a compulsory healthcare plan for all Korean nationals; eligible citizens are covered either through community- or employee-based plan. The health care utilization DB, one of the main databases run by the Service, was used in the present study. The DB holds a vast amount (over 1.5 trillion cases) of inpatient and outpatient data, including diagnosis, length of inpatient admission, type of treatment, and prescription records.

### Study Cohort

The criteria we employed for extracting Ménière disease (MD) cohort from the DB were subjects who (1) had been diagnosed at least twice with KCD (Korean Standard Classification of Diseases) Diagnosis Code ‘H810’, and (2) had undergone pure tone audiometry (PTA, prescription code F6341) on the day of visit. Likewise, atopic dermatitis (AD) cohort was defined as those who (1) had been diagnosed at least twice with KCD Diagnosis code ‘L20’ with any two consecutive visits separated by at least 6 months, and (2) had been prescribed topical calcineurin inhibitors (TCI)-tacrolimus (Protopic®) 0.03% 10 g/0.1% 30 g, or pimecrolimus (Elidel®) 1% 30g-on the day of diagnosis. Vitiligo individuals were defined as those who (1) had been diagnosed with KCD Diagnosis Code ‘L80’, and (2) had been prescribed topical calcineurin inhibitors, topical corticosteroids-methylprednisolone aceponate 1 mg/g (Advantan®) 10 g, prednicarbate 0.025% (Dermatop®) 10 mg, *etc*.-or topical calcipotriol 50 μg/mL (Daivonex®).

### Statistical analysis

A summary of demographic and baseline characteristics was constructed using descriptive analysis; the mean, maximum, minimum and standard deviation (S.D.) for quantitative variables and the frequency and percentage (%) for qualitative variables. Prevalence of atopic dermatitis (AD) and vitiligo, with respect to the status of Ménière disease (MD), was analysed using χ^2^ tests. One of the co-authors, a medical statistician, was tasked with supervision of the overall analytics procedure. All statistical analyses were performed using SAS Enterprise Guide 6.1 M1 (SAS Institute Inc., Cary, NC, United States) and IBM SPSS software package for Windows (version 19.0, Chicago, IL, United States). All tests were two-sided and *p*-values less than 0.05 were deemed statistically significant.

## Data Availability

The datasets presented in the current study are available from the corresponding authors upon request.
